# Serum apoptotic marker M30 is positively correlated with early diastolic dysfunction in adolescent obesity

**DOI:** 10.1371/journal.pone.0217429

**Published:** 2019-05-23

**Authors:** Ming-Chun Yang, Hsien-Kuan Liu, Yu-Tsun Su, Ching-Chung Tsai, Jiunn-Ren Wu

**Affiliations:** 1 Department of Pediatrics, E-Da Hospital, Kaohsiung City, Taiwan; 2 School of Chinese Medicine for Post Baccalaureate, I-Shou University, Kaohsiung City, Taiwan; 3 School of Medicine for International Students, I-Shou University, Kaohsiung City, Taiwan; Universita degli Studi di Roma La Sapienza, ITALY

## Abstract

**Purpose:**

Obesity in adolescence has been shown to be related to cardiac geometric and functional changes. Cardiac dysfunction in adults with obesity could be attributed to chronic low-grade inflammation, apoptosis of cardiomyocyte, and glucose metabolic disorder. The aforementioned association in adolescents with obesity have never been well studied. Our aim was to determine the types of cardiac dysfunction in adolescents with obesity and survey the association between cardiac dysfunction and chronic low-grade inflammation, apoptosis, and glucose dysregulation in adolescents with obesity.

**Methods:**

Adolescents aged between 10 and 20 years were enrolled in this study. Body mass index, waist-to-hip ratio, blood pressure, glucose metabolism, and high-sensitivity C-reactive protein (hs-CRP), interleukin-6 (IL-6), tumor necrosis factor-a (TNF-α), and apoptosis marker M30 levels were measured. Echocardiographic indices were also measured. The association between serum biomarkers and echocardiographic function parameters was analyzed.

**Results:**

Diastolic dysfunction was the major finding in the cardiac functional assessment. The main changes in glucose metabolism were elevated C-peptide level and insulin resistance. Hs-CRP, IL-6, and M30 levels also increased with adolescent obesity. M30 was the major biomarker that was highly correlated to diastolic dysfunction indices in adolescents with obesity.

**Conclusions:**

Diastolic dysfunction was the main change in adolescent obesity. Insulin resistance, apoptotic marker M30, hs-CRP, and IL-6 were all elevated in adolescents with obesity. Only M30 was related to indices of left ventricular diastolic dysfunction among adolescents with obesity, rather than inflammation or insulin resistance.

## Introduction

Obesity is an emerging threat to global health. In Taiwan, the prevalence of obesity in adolescents was 7.3% in 1990. The prevalence of obesity in adolescents nowadays is 16.9%. The prevalence of obesity in male and female adolescents is 20.0% and 13.4% respectively. Although studies in adults documented relationships between obesity and cardiac dysfunction, coexisting diabetes, hypertension, dyslipidemia, renal insufficiency and other chronic diseases could also contribute to the alteration of cardiac functions. Most of the adolescents have no comorbidities, such as diabetes, hypertension, renal insufficiency or autoimmune disorder. This provides reliable research material to assess obesity-related cardiovascular changes. Pediatric obesity leading to alterations of cardiac geometry and diastolic dysfunction has been reported [1–5]. Nevertheless, the results of systolic dysfunction in adolescent obesity were conflicting [1, 2, 4–6]. Left ventricular (LV) dysfunction in adults with obesity could be attributed to impaired coronary flow reserve and coronary microvascular dysfunction [7], chronic low-grade inflammation, apoptosis of cardiomyocyte, and glucose metabolic disorder [8]. However, changes in the aforementioned pathways have never been studied in adolescents with obesity.

High-sensitivity C-reactive protein (hs-CRP) [9, 10], tumor necrosis factor-α (TNF-α) [11], and interleukin-6 (IL-6) [11, 12] have been reported as chronic inflammatory biomarkers associated with cardiac dysfunction in adults. M30 monoclonal antibody level can be used to reflect the degree of apoptosis. During apoptosis, the disintegration of apoptotic cells releases a caspase-cleaved cytokeratin fragment, cytokeratin 18. The M30 antigen is an epitope in cytokeratin 18. Detection of M30 antigen with M30 monoclonal antibody can specifically detect cell apoptosis [13]. This study focused on adolescents aimed to investigate early changes in obesity-related cardiac dysfunction, and to explore the correlations between indices of cardiac dysfunction and serum biomarkers, including hs-CRP, TNF-α, interleukin-6, M30 monoclonal antibody, and insulin resistance.

## Materials and methods

We conducted a cross-sectional study of adolescents. The project was approved by the “Institutional Review Board of the E-DA Hospital” (approval number EMRP25104N). All the informed consents were written and signed by legal guardians and participants themselves.

### Study population and data collection

According to the World Health Organization definition, the age of adolescence is between 10 to 20 years [14]. Participants were recruited voluntarily when they noticed the recruitment poster of this study in E-DA Hospital. The male-to-female ratio was set to be 2:1 since the prevalence of obesity in Taiwanese adolescents is around 2:1. All the participants were in a healthy status. We excluded participants if they had an acute illness or known chronic diseases, including infection, autoimmune disease, previously known heart disease, diabetes mellitus, or kidney disease. The participants were arranged to come to the clinic and underwent checkup for measurement of body height, body weight, waist circumference, hip circumference, and blood pressure (BP). BP was measured with automated office BP measurement, and we recorded a mean of 5 readings with the participants resting quietly and alone. Body mass index (BMI) was recorded as age- and gender-specific BMI based on BMI reference for Taiwanese adolescents [15].

### Group definitions

Adolescents were divided into 3 groups according to BMI percentiles [16]. Those with age- and gender-specific BMI < 95^th^ percentile were classified into group 1 (normal weight). Adolescents with age- and gender-specific BMI ≥ 95^th^ percentile and < 95^th^ percentile x 120% (obesity), and ≥ 95^th^ percentile x 120% (severe obesity) were categorized into groups 2 and 3, respectively.

### Laboratory evaluation and outcome measures

Blood sampling was obtained for fasting glucose, C-peptide, HbA1c, hs-CRP, TNF-α, IL-6, and monoclonal antibody M30 level. Insulin resistance was calculated with the modified homeostasis model assessment of insulin resistance. HOMA-IR (C-peptide) = 1.5 + fasting blood glucose × fasting C-peptide/2800 [17].

Echocardiography was performed in all these healthy adolescents. The purpose of echocardiography was only for study, rather than any medical indications. LV hypertrophy and function were evaluated, including LV mass, LV mass indexed by body height at the 2.7 power (LV mass index) [18], mitral valve inflow deceleration time (MVDT), isovolumic relaxation time (IVRT), isovolumic contraction time (IVCT), LV ejection time (LVET), LV ejection fraction (LVEF), and Tei index. The Tei index, calculated as IVRT plus IVCT divided by LVET, was used to assess the combined systolic and diastolic ventricular functions [19].

Tissue Doppler imaging was obtained at the lateral annulus of the mitral valve to evaluate LV diastolic function. Three measurements at the mitral lateral annulus were averaged to calculate e’, a’, and e’/a’ average. E/e’ is the ratio of early mitral inflow velocity (E) to mitral annular early diastolic velocity (e’). In order to avoid technique and measurement bias, all the echocardiography were performed by the same cardiologist (MC Yang).

### Statistical analysis

Data were presented as mean ± standard deviation (mean ± 1 SD). Analysis of variance (ANOVA) was used to compare the means of independent data in BMI groups 1, 2, and 3. Linear trend was used to demonstrate the trend in the 3 groups with respect to the waist circumference, hip circumference, waist-to-hip ratio, blood pressure, serum biomarkers, insulin resistance, and echocardiographic parameters.

The results of ANOVA analysis showed significant elevations of hs-CRP, IL-6, M30 level, and insulin resistance in adolescents with obesity (group 2 and 3). Multivariate linear regression was further applied to acquire beta coefficient value with corresponding 95% confidence intervals and to assess the linear correlation between LV diastolic function parameters and serum biomarkers in participants with obesity (group 2 and 3). Age, sex, hs-CRP, TNF-α, IL-6, M30 level, and insulin resistance were included in the linear regression models. Collinearity statistics with the variance inflation factor was used to check multicollinearity. If the variance inflation factor is < 5, then multicollinearity is low and the possibility of these variables correlated with each other is also low.

All *p* values were 2-sided, and a *p*-value < 0.05 was regarded as statistically significant. In order to avoid type 1 error in ANOVA analysis, Bonferroni correction was applied to detect possible false positive result.

## Results

**Fig 1:**
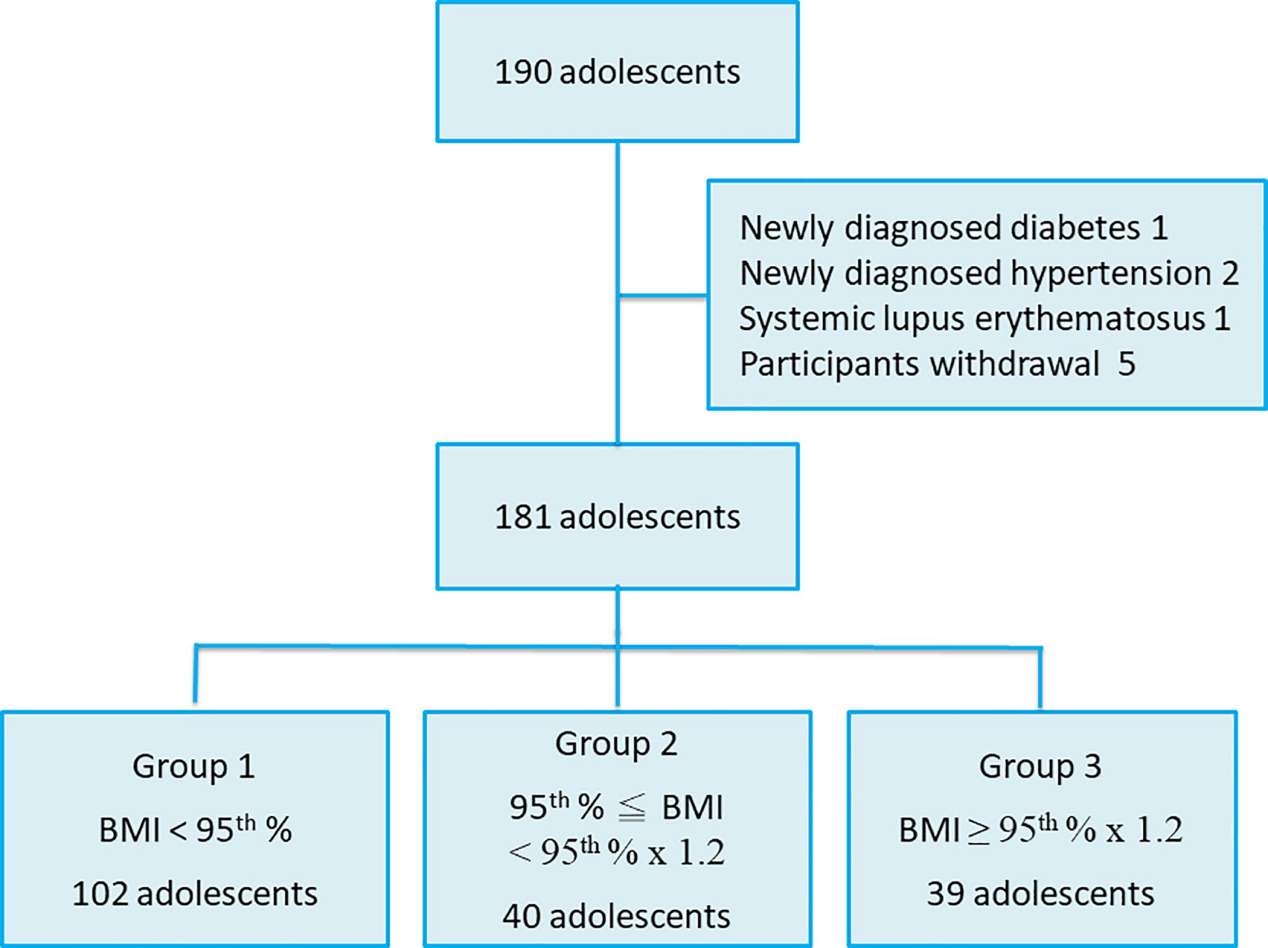
Algorithm and classification of groups.

A total of 190 adolescents participated in this study, and five participants withdrew the consent from the study. Four participants were excluded because of newly diagnosed systemic disease, including severe hypertension (systolic BP > 160 mmHg or diastolic BP > 100 mmHg), diabetes, and systemic lupus erythematosus (Fig 1). There were 181 adolescents enrolled in the study, including 102 participants with normal weight (group 1), 40 participants with obesity (group 2), and 39 participants with severe obesity (group 3) (Fig 1). Age distribution was not significantly different between these 3 groups. The male-to-female ratio was nearly 2:1 in all groups. Waist circumference, hip circumference, waist-to-hip ratio, systolic BP, and diastolic BP increased significantly in accordance with the BMI groups (*p* < 0.001) (Table 1). Bonferroni corrections also confirmed these correlations.

**Table 1:** Demographic characteristics of age, waist circumference, hip circumference, blood pressure, glucose metabolism, and serum biomarkers.

	All	Group 1	Group 2	Group 3	*P* value of ANOVA	*P* trend
BMI percentile		< 95^th^	≥ 95^th^—< 95^th^ x 120%	≥ 95^th^ x 120%		
No. of participants	181	102	40	39		
Age (year)	13.66 ± 2.52	13.78 ± 2.47	13.48 ± 2.71	113.56 ± 2.49	0.79	0.65
M/F	120/61	66/36	27/13	27/12	0.58	0.78
Waist circumference (cm)	78.82 ± 16.29	67.44 ± 8.09	85.96 ± 6.86	101.45 ±± 10.17	< 0.001	< 0.001
Hip circumference (cm)	93.17 ± 12.96	85.32 ± 8.21	97.91 ± 8.50	108.99 ± 9.74	< 0.001	< 0.001
Waist/hip ratio	0.84 ± 0.09	0.79 ± 0.06	0.88 ± 0.07	0.93 ± 0.05	< 0.001	< 0.001
Systolic BP (mmHg)	116 ± 13	111 ± 11	120 ± 13	127 ± 12	< 0.001	< 0.001
Diastolic BP (mmHg)	72 ± 10	70 ± 9	75 ± 9	76 ± 10	< 0.001	< 0.001
**Glu**cose metabolism						
Fasting glucose (mg/dL)	94 ± 8	95 ± 7	94 ± 7	92 ± 9	0.15	0.06
HbA1c (%)	5.39 ± 0.28	5.34 ± 0.25	5.49 ± 0.21	5.39 ± 0.36	0.05	0.44
C-peptide (ng/mL)	2.59 ± 1.66	1.65 ± 0.51	2.50 ± 0.72	4.17 ± 2.21	< 0.001	< 0.001
HOMA-IR	1.90 ± 1.08	1.23 ± 0.39	1.86 ± 0.55	2.99 ± 1.29	< 0.001	< 0.001
**Serum biomarke**rs						
Hs-CRP (mg/L)	2.01 ± 2.71	0.69 ± 0.96	2.23 ± 2.22	3.90 ± 3.72	< 0.001	< 0.001
TNF-± (pg/mL)	7.08 ± 15.28	8.80 ± 19.50	7.19 ± 11.48	3.46 ± 5.31	0.31	0.13
IL-6 (pg/mL)	3.31 ± 3.44	2.60 ± 2.77	2.45 ± 2.77	5.55 ± 4.28	< 0.001	< 0.001
M30 level (U/L)	147 ± 146	77 ± 47	206 ± 154	233 ± 197	< 0.001	< 0.001

Data presented as mean ± 1 SD; M/F, male-to-female ratio; HOMA-IR, homeostasis model assessment of insulin resistance; hs-CRP, high-sensitivity C-reactive protein; TNF-α, tumor necrosis factor-α; IL-6, interleukin-6

ANOVA was used to compare means in groups 1, 2, and 3

### Correlations between obesity, glucose metabolism, and serum biomarkers

In glucose metabolism, C-peptide level and insulin resistance (HOMA-IR) increased significantly in accordance with the BMI groups (*p* < 0.001) (Table 1). HbA1c and fasting sugar did not differ in the 3 groups.

The range of hs-CRP was from 0.1 to 18.01 mg/L. There were 7 participants whose hs-CRP were < 0.10 mg/L, and their data were recorded as 0. IL-6 ranged from 0.05 to 15.47 pg/mL. There were 20 participants with undetectable IL-6 (< 0.05 pg/mL), and their IL-6 were recorded as 0. The range of TNF-α was from 0.1 to 106.88 pg/mL. There were 29 participants with undetectable TNF-α (< 0.10 pg/mL), and their TNF-α was recorded as 0. M30 level ranged from 8.88 to 948.93 U/L. Hs-CRP, IL-6, and apoptotic marker M30 levels increased in accordance with the BMI groups (*p* < 0.001) (Table 1). TNF-α level did not show differences between the 3 groups. The results implied that adolescent obesity was related to dysregulated glucose metabolism, chronic inflammation, and cell apoptosis.

### Correlations between obesity, LV mass, and LV function

LV mass and LV mass index were positively correlated with the BMI groups (*p* < 0.001). Obesity correlated well with LV diastolic dysfunction but not with systolic dysfunction or Tei index (Table 2). Mitral inflow E/A ratio tended to be negatively correlated with BMI groups (*p* = 0.001). Tissue Doppler imaging also confirmed LV diastolic dysfunction in adolescents with obesity. Mitral lateral annulus e’ velocity and mitral lateral annular e’/a’ ratio were negatively correlated with BMI groups, respectively (*p* < 0.001). E/e’ ratio at the mitral lateral annulus, which was positively correlated with LV end-diastolic pressure [20], was positively correlated with BMI groups (*p* < 0.001). Bonferroni corrections also confirmed these correlations. LV ejection fraction and Tei index were similar between the 3 BMI groups. This showed that adolescent obesity was associated with LV hypertrophy and higher LV end-diastolic pressure.

**Table 2:** Comparisons of LV mass and LV function between the 3 groups.

	All	Group 1	Group 2	Group 3	P-value of ANOVA	Linear trend
**N**	181	102	40	39		
**LV mass**						
LV mass (g)	146 ± 60	116 ± 37	162 ± 47	212 ± 65	< 0.001	< 0.001
LV mass index (g/m^2.7^)	41.29 ± 12.91	34.12 ± 8.45	44.87 ± 8.36	56.66 ± 11.69	< 0.001	< 0.001
**LV systolic and diastolic function**						
E/A ratio	2.07 ± 0.51	2.17 ± 0.51	2.04 ± 0.49	1.81 ± 0.43	0.001	< 0.001
Mitral lateral e’ (cm/s)	16.38 ± 2.86	17.39 ± 2.60	16.23 ± 2.32	13.82 ± 2.41	< 0.001	< 0.001
Mitral lateral e’/a’ ratio	2.51 ± 0.75	2.79 ± 0.72	2.31 ± 0.63	1.95 ± 0.51	< 0.001	< 0.001
Mitral E/e’ (lateral)	6.44 ± 1.51	5.98 ± 1.17	6.63 ± 1.27	7.47 ± 1.97	< 0.001	< 0.001
MVDT (ms)	191 ± 43	187 ± 38	192 ± 47	202 ± 50	0.15	0.05
IVRT (ms)	56 ± 13	55 ± 13	56 ± 14	60 ± 13	0.15	0.05
IVCT (ms)	55 ± 18	55 ± 19	51 ± 15	56 ±17	0.40	0.75
LVET (ms)	288 ± 23	288 ± 24	291 ± 20	286 ± 25	0.65	0.71
LVEF (%)	68 ± 6	68 ± 6	69 ± 6	68 ± 7	0.35	0.62
Tei index	0.39 ± 0.09	0.38 ± 0.09	0.37 ± 0.08	0.41 ± 0.10	0.18	0.14

Data presented as mean ± 1 SD; LV, left ventricle; MVDT, mitral valve inflow deceleration time; IVRT, isovolumic relaxation time; IVCT, isovolumic contraction time; LVET, LV ejection time; LVEF, left ventricular ejection fraction

ANOVA was used to compare means in groups 1, 2, and 3

### Correlations between serum biomarkers and indices of LV diastolic dysfunction

Since the relationship between adolescent obesity and LV diastolic dysfunction was established. Further research on the association between serum biomarkers and diastolic function indices was conducted. In all participants with obesity (group 2 and 3), correlations between serum biomarkers (hs-CRP, IL-6, TNF-α, M30 level, insulin resistance) and LV diastolic function parameters were analyzed by multivariate regression (Table 3). Mitral lateral annulus diastolic velocity e’ was negatively correlated with M30 level (*p* = 0.03) and insulin resistance (*p* < 0.01). High apoptotic marker M30 level and high insulin resistance were associated with slower mitral lateral annular diastolic velocity. Mitral lateral annulus e’/a’ ratio was negatively correlated with M30 level (*p* = 0.03). The E/e’ of mitral lateral annulus was positively correlated with M30 level (*p* = 0.02), which represented that elevated M30 level was associated with elevated LV end-diastolic pressure. Collinearity statistics with the variance inflation factor showed the possibility of these variables correlated with each other was low.

**Table 3:** Multivariate regression of LV diastolic function indices and serum biomarkers in participants with obesity.

	Unstandardized ß-coefficients	Standardized ß-coefficients	95% CI	*P*-value	Collinearity statistics (VIF)
**Mitral lateral e’**					
M30 level (U/L)	-0.004	-0.271	-0.008–0.000	0.03	1.04
HOMA-IR	-0.934	-0.381	-1.56 –-0.308	< 0.01	1.13
Hs-CRP (mg/L)	-0.023	-0.028	-0.236–0.190	0.83	1.18
IL-6 (pg/mL)	-0.135	-0.197	-0.312–0.043	0.13	1.16
TNF-α (pg/mL)	0.002	0.005	-0.071–0.074	0.97	1.06
**Mitral lateral e’/a’**					
M30 level (U/L)	-0.001	-0.293	-0.002–0.000	0.04	1.04
HOMA-IR	-0.060	-0.110	-0.215–0.096	0.45	1.09
Hs-CRP (mg/L)	0.011	0.061	-0.042–0.064	0.68	1.18
IL-6 (pg/mL)	-0.019	-0.128	-0.063–0.025	0.38	1.16
TNF-α (pg/mL)	0.001	0.010	-0.017–0.019	0.94	1.04
**E/e’ (mitral lateral annulus)**					
M30 level (U/L)	0.003	0.303	0.000–0.005	0.03	1.04
HOMA-IR	0.037	0.025	-0.375–0.448	0.86	1.13
Hs-CRP (mg/L)	-0.049	-0.101	-0.189–0.091	0.48	1.18
IL-6 (pg/mL)	0.098	0.240	-0.019–0.215	0.10	1.16
TNF-α (pg/mL)	0.015	0.084	-0.033–0.062	0.54	1.06

VIF, The Variance Inflation Factor; HOMA-IR, homeostasis model assessment of insulin resistance; HsCRP, high sensitivity C-reactive protein; IL-6, interleukin-6; TNF-α, tumor necrosis factor-α

## Discussion

In this study, certain important findings were drawn. Glucose metabolism dysregulation, which was reflected in C-peptide and insulin resistance, could be observed in adolescents with obesity. Chronic inflammation markers (hs-CRP and IL-6) and apoptotic marker M30 level were all positively correlated to the degree of obesity. LV diastolic dysfunction was the main change in adolescents with obesity. Apoptotic biomarker M30 level was significantly correlated with LV diastolic dysfunction in this study. There have been previous adolescent studies showing that obesity is correlated with LV hypertrophy [21]. Increased LV mass could be linked to hemodynamic and metabolic pathways. Change in cardiac output, obesity-related obstructive sleep apnea, insulin resistance, and adipokines are all responsible for LV hypertrophy [22]. Diastolic dysfunction in adolescents with obesity was the major finding of cardiac functional alteration in our study. Previous childhood and adolescence studies also revealed LV diastolic dysfunction in participants with obesity [23, 24]. In adolescents with morbid obesity, LV diastolic dysfunction and LV hypertrophy were reversible after weight reduction [25].

LV diastolic dysfunction was regarded as a preceding manifestation of systolic failure and diabetic cardiomyopathy [26]. In adult studies, LV diastolic dysfunction can be attributed to cardiomyocyte apoptosis, coronary artery disease, chronic inflammation, and insulin resistance. We found that apoptosis marker M30 level, rather than chronic inflammation biomarkers or insulin resistance, was highly associated with diastolic functional deterioration in adolescents with obesity. Cardiomyocyte apoptosis has been proved to be involved in embryonic heart development [27]. Apoptotic cardiomyocytes have been identified in the trabecular and compact zones of the mouse ventricles from embryonic days to early birth days. Embryonic outflow tract shortening is a normal process at specific stages of cardiac development, which is related to myocyte apoptosis [28]. If apoptosis is involved in abnormal cardiac morphogenesis, it may also lead to congenital heart malformations [29].

Cardiomyocyte apoptosis is also related to the aging process and chronically overloaded heart [30]. Apoptosis plays a significant role in cardiac geometric change and progressive worsening in myocardial function, which may lead to chronic cardiomyopathy and end-stage heart failure [31, 32]. Apoptosis marker M30 level has been shown to be an independent predictor of LV remodeling and dysfunction in elderly patients with ST-segment elevation myocardial infarction [33]. Cardiomyocyte apoptosis can be induced by hypoxia and ischemia [34, 35], and we noticed that it was also correlated to adolescents with obesity and diastolic dysfunction.

We also found a weak correlation between insulin resistance and the index of LV diastolic dysfunction. Only mitral lateral annulus diastolic velocity e’ was correlated with insulin resistance. Although the LV diastolic dysfunction was not highly associated with insulin resistance, insulin resistance remained an important finding in adolescents with obesity itself. Obesity is an important risk factor for heart failure in adults, which has been considered to be partly mediated by insulin resistance [36]. Advanced glycosylation products could result in myocardial stiffness [37]. Hyperinsulinemia was also related to myocardium growth, sodium retention, volume extension, and subsequent myocardial dysfunction [38].

Elevation of IL-6 level was linked to LV diastolic dysfunction [12]. Our study found that IL-6 level was significantly elevated in adolescents with obesity. However, IL-6 was not related to diastolic dysfunction in adolescent obesity. Hs-CRP has been shown to be associated with LV dysfunction in adult studies [9, 10]. Research showed that hs-CRP was related to plaque remodeling, oxidative stress, endothelial dysfunction, prothrombotic state, activation of complement, and vascular wall damage [39]. These mechanisms might account for diastolic dysfunction in obesity. Nevertheless, our analysis revealed that hs-CRP was not related to LV diastolic dysfunction in adolescents with obesity.

## Limitations

We found changes in diastolic parameters in adolescents with obesity. These changes in diastolic function were subclinical. Childhood studies found higher E/e’ and lower e’/a’ in children with hypertension [40], and hypertrophic cardiomyopathy [41]. However, there is no cutoff value for E/e’ or lateral e’ velocity to indicate diastolic dysfunction in children and adolescents.

Furthermore, changes in serum biomarkers only represented its association with cardiac dysfunction. The causality between M30 level and LV diastolic dysfunction remains unclear. There was no previous cardiac function assessment in our participants. A prospective cohort study to follow up cardiac functional alterations and changes in M30 level in these participants is a more reliable method to prove cardiac function changes and possible mechanisms leading to the cardiac dysfunction in adolescents with obesity. This provides further study materials in the future.

## Conclusions

LV diastolic dysfunction and LV hypertrophy were the main changes in adolescent obesity. Insulin resistance, apoptotic marker M30 level, hs-CRP, and IL-6 were all elevated in adolescents with obesity. Only M30 level was highly correlated to LV diastolic function indices in adolescents with obesity. There was a weak correlation between insulin resistance and the index of LV diastolic dysfunction. Although the LV diastolic dysfunction was not highly associated with insulin resistance, insulin resistance was still an important finding in adolescents with obesity.
